# Two new Korean earthworms (Annelida, Oligochaeta, Megadrilacea, Megascolecidae)

**DOI:** 10.3897/zookeys.307.5362

**Published:** 2013-06-06

**Authors:** Robert J. Blakemore, Seunghan Lee, Wonchoel Lee, Hong-Yul Seo

**Affiliations:** 1 Biodiversity Laboratory, College of Natural Science, Hanyang Uni., Seoul 133-791; 2 National Institute of Biological Resources (NIBR), Incheon 404-708, Korea

**Keywords:** Soil fauna, invertebrate biodiversity, new endemic taxa, molecular barcodes

## Abstract

Two Korean endemic pheretimoid *Amynthas* Kinberg, 1867 species belonging in family Megascolecidae s. stricto are sketched, dissected and described. *Amynthas daeari* Blakemore **sp. n.** has spermathecae in 6/7/8 complying with an *Amynthas tokioensis* spp-group, whilst *Amynthas jinburi* Blakemore **sp. n.** has spermathecal pores in 5 & 6 strictly complying with Sims and Easton’s (1972) *Amynthas canaliculatus*-group. A definitive COI gene barcode is provided for the holotype of *Amynthas daeari* but the age since collection or preservation of the *Amynthas jinburi* type in 2000 precluded its mtDNA extraction at this time.

## Introduction

Specimens from the collection of NIBR contribute to ongoing earthworm surveys as part of understudied non-insect invertebrates of the Korean Peninsula (Blakemore 2012a, b, 2013a, b, Blakemore et al. 2012a, b). Two specimens are described below belonging to a pheretimoid (*Pheretima* auct.) group of Oriental origin that provides approximately 970 valid species from 1,200 nominal taxa (Blakemore 2008a). *Amynthas* Kinberg, 1867 is the most diverse of the pheretimoid genera with some species common to Korea and Japan where faunal totals both approach 100 megadrile earthworm taxa (Blakemore 2003, 2008b, c, 2012c, d). The probable endemics are here added to the Korean list.

## Materials and methods

Taxonomic determinations follow the methodology and classifications in Sims and Easton (1972) and Blakemore (2010). Abbreviations are: C – circumference, GMs – genital markings, lhs – left hand side and rhs – right hand side.

Specimens, now in 80% Ethanol, are lodged in the NIBR facility. Small tissue samples taken for mtDNA COI barcoding as proposed 10 yrs ago by Hebert et al. (2003) used similar methods as per Blakemore et al. (2010) with preliminary analysis via BLAST programs (www.blast.ncbi.nlm.nih.gov/BLAST.cgi).

Discussion is confined to remarks after each species’ description. For brevity, not all taxonomic authorities are cited in References as these may be sought elsewhere.

## Taxonomic results

### Annelida Lamarck, 1802 Oligochaeta Grube, 1850 Megadrilacea Benham, 1890 Megascolecidae Rosa, 1891 *sensu* Blakemore, 2000 *Amynthas* Kinberg, 1867

#### Amynthas daeari sp. n.

urn:lsid:zoobank.org:act:E6B103F4-2FFE-4A05-BCD8-356A90754AC5

http://species-id.net/wiki/Amynthas_daeari

##### Material examined.

IV0000261261, mature specimen complete but broken in two at clitellum after being figured and dissected. Collected from small valley at Jeollabuk-do, Wanju-gun, Dongsang-myeon, Daea-ri (35.9801N, 127.2981E); collected 27^th^ July, 2012 by Dr Hong-Yul Seo. DNA tissue sample code – w53.

##### Etymology.

Noun from location.

##### Diagnosis.

*Amynthas* with two pairs of spermathecal pores in 6/7/8 complying with an *Amynthas tokioensis*-group; spermathecae with compressed clavate diverticula; GMs median to spermathecal and male pores with patches around the former and the latter bracketed laterally by small C-shaped clefts.

##### Distribution.

Only known from a single specimen from type locality.

##### Habitat.

In litter layer in forest.

**Behaviour.** Habitat, pigmentation and gut contents indicate activity in the litter layer.

##### Description.

**Length.** 150 mm.

**Width.** ca. 7 mm at male pore level.

**Segments.** 107.

**Colour.** Brown in alcohol, possibly darker in life as liquid was stained.

**Prostomium.** Open epilobous.

**First dorsal pore.** 12/13.

**Setae.** Ca. 60 per segment, approximately 22-24 between spermathecal and male pores.

**Nephropores.** Not found.

**Clitellum.** Annular 14-16, setae occluded.

**Male pores.** On 18 centred on small, round porophore (found by following a pin from prostate gland exit) with GMs anterio-median and shallow clefts laterally (that function as seminal ducts and/or suction cups?).

**Female pores.** Single on 14.

**Spermathecal pores.** 6/7/8 ca 0.3 C apart at edge of puckered area and lateral to GMs.

**Genital markings.** Paired discs just median to male and spermathecal pores as noted; composite glands on spermathecal pore GMs but none found for GMs near male pores although the body here is macerated and they may well have broken off and dissipated.

**Septa.** Nephridial forests on septa 5 & 6; 7/8 thin, 8/9/10 aborted.

**Dorsal blood vessel (dbv).** Single.

**Hearts.** Last hearts in 13 (preceding vascularization unclear/damaged).

**Gizzard.** Single in 8-9.

**Calciferous glands.** Absent.

**Intestine.** Indeterminate as specimen macerated; caeca ventrally incised from 27; typhlosole not noted.

**Nephridia.** Meroic.

**Male organs.** Holandric, seminal vesicles in 11 & 12.

**Ovaries.** In 13 as usual.

**Prostates.** Racemose glands in 17-19, duct short and muscular.

**Spermathecae.** Two pairs in 7 and 8; that in 7lhs inflated, that in 8lhs deflated (showing how meaningless such a distinction is although relied on by some authors).

**Gut contents.** Coarse organic debris, i.e., a litter diet suggesting superficial feeding.

##### DNA COI barcode.

>w53 *Amynthas daeari* Holotype.

CTATATTTCATTTTAGGAATTTGAGCTGGAATAATTGGGGCAGGAATAAGACTGCTTATTCGAATTGAGCTAAGACAGCCGGGCTCTTTTCTAGGAAGGGATCAACTCTATAATACAATTGTAACAGCTCATGCATTTTTAATAATCTTCTTTCTTGTAATACCAGTATTTATTGGTGGGTTTGGAAATTGACTTCTACCTCTAATACTAGGTGCCCCAGATATAGCTTTCCCGCGACTTAACAATATAAGATTCTGATTACTGCCCCCATCACTAATTTTACTAGTATCGTCTGCAGCAGTAGAAAAAGGTGCCGGAACAGGATGGACAGTGTACCCCCCACTTGCGAGAAACATTGCACATGCCGGCCCTTCAGTAGATCTTGCAATTTTTTCTCTCCATCTAGCCGGAGCATCATCAATTCTCGGTGCCATCAACTTCATTACTACCGTAATTAATATACGATGATCTGGGCTACGCTTAGAACGAATTCCTCTATTTGTATGAGCAGTTGTAATTACTGTAATTCTTTTACTTCTATCTTTACCAGTCTTAGCCGGTGCTATTACAATATTACTAACAGACCGAAACCTAAATACATCATTTTTTGATCCAGCGGGAGGAGGTGATCCAATTCTATATCAACACTTATTT

megaBLAST result: “ *Amynthas tappensis*” (AB542547.1) from Japan max. identity <88% this then is a different and likely new taxon. The closest match from current Korean studies with BLASTn identity 565/653 (87%) is WO49, an immature *Amynthas* sp. from Jeju that itself comes closest to the *Amynthas tokioensis*/*Metaphire hilgendorfi* spp. complexes (see Blakemore 2013a: Appendix).

##### Remarks.

Of *Amynthas* species with spermathecae in 6/7/8, twenty or so in the *Amynthas tokioensis*-group of Sims and Easton (1972) mostly have manicate caeca, such as *Amynthas kanrazanus* (Kobayashi, 1937); about twenty other species, many placed in this group after 1972, have simple intestinal caeca. Only four have simple incised caeca as here, but they all differ in characteristics of their GMs, at least, and none of these latter are known from Korea (Blakemore unpublished). The incised caeca is assumed to be a characteristic transitional or intermediate from simple to complex/manicate. The GMs in 7-8 obviously correspond to those in 18 during amphimixis but it is not known whether they interlock serially. The shape of the spermathecae and spermathecal pores are further distinguishing characteristics of *Amynthas daeari* that, along with its objective DNA barcode data, now serve to define this taxon.

**Figure 1.**
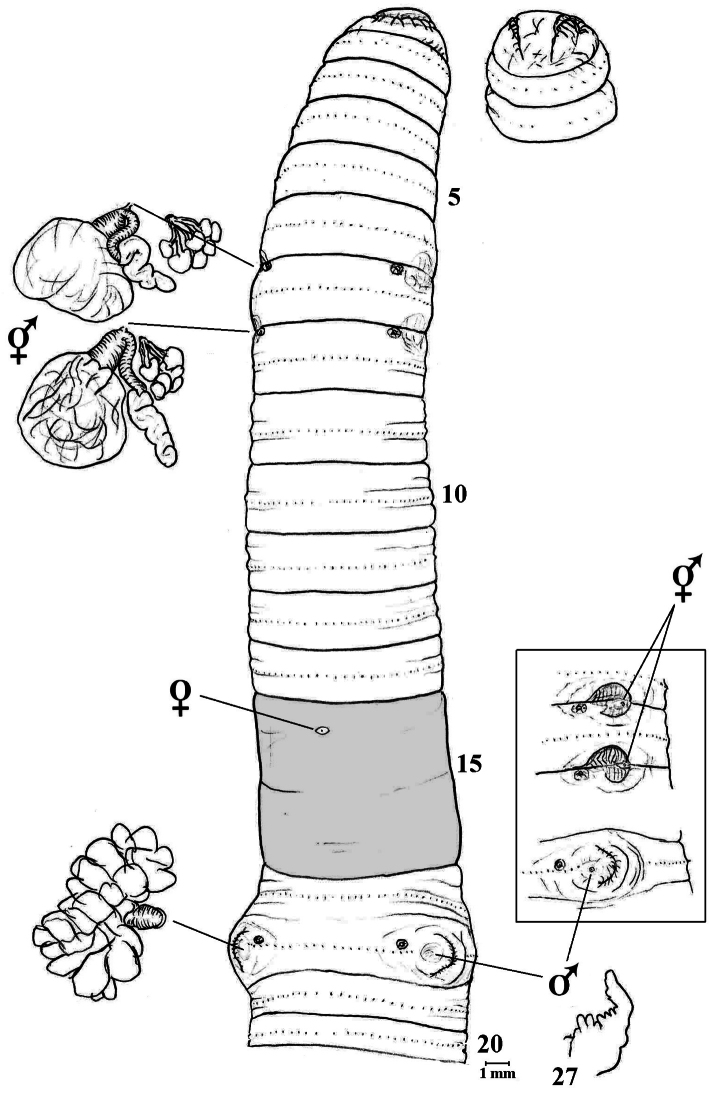
*Amynthas daeari* sp. n. showing ventral view with spermathecae, their composite genital marking glands and 18lhs prostate *in situ* plus incised intestinal caecum in 27; dorsal view of prostomium; [boxed are lateral views of spermathecal pores and male field in 18rhs to same scale].

#### Amynthas jinburi sp. n.

urn:lsid:zoobank.org:act:9BA5299B-0BA0-4E50-8EAC-C76C2D66B2EA

http://species-id.net/wiki/Amynthas_jinburi

##### Material examined.

IV0000213690, sub-mature specimen, figured and dissected. From Gangwon-do, Goseong-gun, Ganseong-eup, Jinbu-ri (ca. 38.2961N, 128.3546E) just north of Seoraksan Park on East coast; collected 1^st^ – 2^nd^ June, 2000 by unknown person(s) and deposited in NIBR. DNA tissue sample w61b (unsuccessful at this time).

##### Etymology.

Noun from location.

##### Diagnosis.

*Amynthas* with two pairs of spermathecal pores in 5 & 6; long, clavate spermathecal diverticula; simple caeca; and GMs absent except for large patches surrounding male pores.

##### Distribution.

Known only from single specimen from type locality.

##### Habitat.

Jinburi is a remote, mountainous and forested area

**Behaviour.** Possibly deep burrowing and geophageous (from gut contents).

##### Description.

**Length.** 210 mm.

**Width.** ca. 10 mm at male pore level.

**Segments.** 143 with some secondary annulation (from preservation?).

**Colour.** Bleached pale yellow in aged alcohol, possibly darker in life.

**Prostomium.** Open epilobous.

**First dorsal pore.** 11/12.

**Setae.** >100 per segment; e.g. 100+ on 11 and 112 counted on segment 12; approximately 16 setae intervene between male pore pads that are asetal on 18.

**Nephropores.** Not found.

**Clitellum.** Slightly darker at 14-16.

**Male pores:** On 18 on small, rounded and flat porophores.

**Female pores.** Single on 14.

**Spermathecal pores.** At posterior of 5 and 6 approximately 0.3 C apart.

**Genital markings.** None (sub-adult?).

**Septa.** Nephridial forests on septa 5 & 6; 5/6/7/8 thick, 8/9 thin to base of gizzard, 9/10 aborted.

**Hearts.** Seen in 11-13 (aborted in 10?).

**Gizzard.** Single in 8-9.

**Calciferous glands.** Absent.

**Intestine.** From 15; caeca simple elongate from 27; typhlosole not noted.

**Nephridia.** Meroic.

**Male organs.** Holandric, testes small in 10 &11; seminal vesicles in 11 & 12.

**Ovaries.** Compact in 13; ovisacs not found in 14.

**Prostates:** Racemose glands not fully developed in 18 on short, muscular duct.

**Figure 2.**
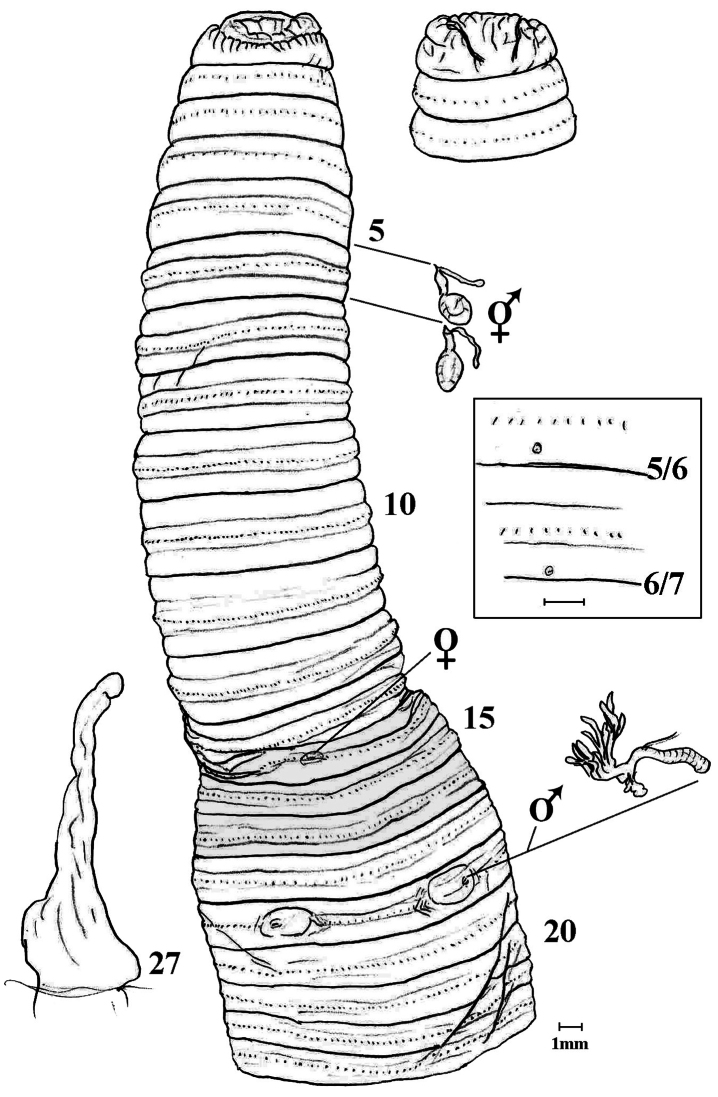
*Amynthas jinburi* sp. n. showing ventral view with spermathecae and 18rhs prostate *in situ* plus simple intestinal caecum in 27; dorsal view of prostomium; [boxed is 2X lateral view of spermathecal pores in 5 & 6rhs].

**Spermathecae.** Two pairs in 6 & 7 exiting to anterior of 5/6 and 6/7 in 5 & 6 (Fig. 2).

**Gut contents.** Filled with yellow soil, i.e. probably a deep-burrowing subsoil geophage.

##### DNA COI barcode.

>w61b– nil result, DNA not extractable on this older material that may have been fixed in formalin (although there was no odour) or denatured by pH.

##### Remarks.

Of all 970 pheretimoid species (Blakemore 2008a), only two are known to have spermathecal pores in 5 & 6: viz. *Amynthas serenus* (Gates, 1936) from Pahang, Malaysia that also lacks GMs, and *Amynthas? breviclitellatus* (Do & Tran, 1995) from Vietnam that differs, at least, in its GMs in 7, 18 and 19. From “Kôryô” Korea (about 30 Km from Seoul), *Amynthas fibulus fibulus* (Kobayashi, 1936: 159) is superficially similar but has spermathecal pores anteriorly in 6 & 7 (rather than posteriorly in 5 & 6) plus its caeca are incised ventrally (rather than smooth); ditto for *Amynthas fibulus ranunculus* (Kobayashi, 1936: 162) that further has slits lateral to male pores. Interestingly, Kobayashi’s (1936: fig. 6) sketch of a prostate gland of *Amynthas fibulus* closely resembles the current specimen’s gland (Fig. 2).

It should be here noted that Sims and Easton (1972) inadvertently place these two *fibulus* taxa in an *Amynthas morrisi*-group defined with spermathecae in 5/6/7 despite Kobayashi (1936: 159) stating “ *Spermathecal pores*, *minute, 2 pairs anteriorly located on VI and VII, closely to the intersegmental furrows*”, i.e. strictly complying with Sims & Easton’s *canaliculatus*-group (then comprised of *benignus* Chen, 1946; *canaliculata* Gates, 1932; *ralla* Gates, 1936: 104; and *rallida* Gates, 1936: 106). It appears that many of Hong and James’ (2001: 67, 68, 69, 75) taxa have a similar attribute although their descriptions are ambiguously stated, such as: “ *Spermathecal pores in 5/6 and 6/7...at or near leading edge of vi, vii*” and no useful figures are provided for the reader to decide.

If spermathecal pores were in 5/6/7 in any of the above taxa, then the *morrisi*-group’s possible nearest relatives from Korea would likely be *Amynthas koreanus* (Kobayashi, 1938: 115) that, however, has manicate caeca; or *Amynthas kobayashii* (Kobayashi, 1938: 119) and *Amynthas geojeinsulae* (Song & Paik, 1970) that both have male fields from 17-19 but differ in simple or incised caeca, respectively; or *Amynthas assimilis* Hong & Kim, 2002 that, like many of its similar cited taxa, has seminal grooves on 18.

The current species has simple, superficial male pores on large disc-like pads on 18. Although not fully mature, it appears unique in the Korea fauna on its combination of this aspect of its male field, spermathecal pores in 5 & 6 and its profusion of setae that number more than 100 per segment, combined with simple elongate intestinal caeca.

Fresh topotypic material is required to confirm these conclusions and to provide definitive DNA data, unless refinement of techniques allows extraction from older types.

## Supplementary Material

XML Treatment for Amynthas daeari

XML Treatment for Amynthas jinburi
